# A Preliminary Incision

**Published:** 1899-02-04

**Authors:** 


### A PRELIMINARY INCISION.

Lecturing at St. Bartholomew's Hospital on cancer of the breast, Mr. Butlin[1] made some interesting observations as to the advantages of making a preliminary incision before proceeding to the removal of a presumedly cancerous breast whenever there is the least doubt as to the nature of the tumour. The observance of this rule has, he says, saved many mistakes, and prevented the performance of larger operations than have been necessary. Even an incision, however, will not always prevent error, and he has often been astonished at the confidence which some surgeons have in the possibility of deciding on the nature of a disease felt or dimly seen through an incision. Several times of late years, where an incision has left him in doubt, Mr. Butlin has cut out a portion of the tumour for microscopical examination, closed the wound, and amputated at the end of a week or ten days if the disease has proved to be carcinoma. Of the desirability of this preliminary incision he has seen more than one excellent proof. "Many years ago," he says, "when I was constantly engaged in examining tumours which had been removed by other people, an old pupil of the hospital sent me a breast which he and his senior partner had removed for cancer. They sent it to me just as it had been removed. Before cutting into the tumour I felt it, and thought that it contained fluid. I then cut into it, when lo! a quantity of clear fluid ran out, and the 'cancer' disappeared. I was shocked at the time to think so needless a mutilation had been practised for the cure of a simple cyst; and I wrote to the operator telling him what had been found, and advising him in future to make an incision into a tumour before he began to remove it, as we frequently do here at the hospital. At the same time, I advised him that he should keep his own counsel, as the mischief had been done, and there was no means of remedying it; that he should not even tell his partner, who did not operate himself, and the knowledge of the mistake was not likely to be useful to him. I also told my friend that he would certainly achieve a reputation which he did not deserve for curing cancer of the breast by operation. This, I am grieved to say, has come quite true; for not long ago I met the senior partner, and asked him whether he remembered this woman, and whether she was yet alive. On which he rubbed his hands, and said, 'Of course I do. She is perfectly well, and the cancer has never returned, although it is many years since the operation was performed.'" The lesson thus taught by Mr. Butlin is one which is very valuable at the present day, in view of the large operations which are now performed for the removal of cancer. In days gone by, when the operation itself was but a small affair, an error did but lead to an unnecessary mutilation—bad enough, but nothing serious to life. At the present day, however, a complete operation, although, perhaps, not a very serious affair compared with some things which are done on the human body, is still a large operation involving a not inconsiderable risk, and it becomes more than ever important not to operate till one knows.

[1] St. Bartholomew's Hosp. Jour.

